# 3D-MiXD: 3D-printed X-ray-compatible microfluidic devices for rapid, low-consumption serial synchrotron crystallography data collection in flow

**DOI:** 10.1107/S2052252519016865

**Published:** 2020-01-16

**Authors:** Diana C. F. Monteiro, David von Stetten, Claudia Stohrer, Marta Sans, Arwen R. Pearson, Gianluca Santoni, Peter van der Linden, Martin Trebbin

**Affiliations:** a The Hamburg Centre for Ultrafast Imaging, Universität Hamburg, Luruper Chaussee 149, 22761 Hamburg, Germany; b Hauptman–Woodward Medical Research Institute, 700 Ellicott Street, Buffalo, NY 14203, USA; c European Molecular Biology Laboratory, Notkestrasse 85, 22607 Hamburg, Germany; d The Astbury Centre for Structural Molecular Biology, University of Leeds, Leeds LS2 9JT, England; e Department of Physics, Universität Hamburg, Jungiusstrasse 9, 20355 Hamburg, Germany; f European Synchrotron Radiation Facility, 71 Avenue des Martyrs, CS 40220, 38043 Grenoble, France; g Partnership for Soft Condensed Matter, European Synchrotron Radiation Facility, 71 Avenue des Martyrs, CS 40220, 38043 Grenoble, France; h Department of Chemistry, The State University of New York at Buffalo, Natural Sciences Complex 760, Buffalo, NY 14260-3000, USA

**Keywords:** serial synchrotron crystallography, microfluidics, 3D printing, 3D microfabrication, 3D-MiXD, structure determination

## Abstract

Serial crystallography has enabled the study of complex biological questions through the determination of biomolecular structures at room temperature using low X-ray doses. Furthermore, it has enabled the study of protein dynamics by the capture of atomically resolved and time-resolved molecular movies. However, the study of many biologically relevant targets is still severely hindered by high sample consumption and lengthy data-collection times. By combining serial synchrotron crystallography (SSX) with 3D printing, a new experimental platform has been created that tackles these challenges. An affordable 3D-printed, X-ray-compatible microfluidic device (3D-MiXD) is reported that allows data to be collected from protein microcrystals in a 3D flow with very high hit and indexing rates, while keeping the sample consumption low. The miniaturized 3D-MiXD can be rapidly installed into virtually any synchrotron beamline with only minimal adjustments. This efficient collection scheme in combination with its mixing geometry paves the way for recording molecular movies at synchrotrons by mixing-triggered millisecond time-resolved SSX.

## Abstract 2

A 3D-printed, X-ray-compatible microfluidic device (3D-MiXD) has been developed and used to rapidly collect highly redundant diffraction data sets from protein microcrystals in flow using a monochromatic serial synchrotron crystallography approach.

## 1. Introduction   

Serial synchrotron crystallography (SSX) is a data-collection approach in which diffraction data are collected from low-millisecond X-ray exposures of protein microcrystals. One of the main advantages of this technique is the low X-ray dose that is accumulated by the crystals during data collection, as fresh crystalline material is available for each new exposure (Ebrahim *et al.*, 2019 ▸; Owen *et al.*, 2017 ▸). The sample refreshment also allows data to be collected at room temperature, eliminating any structural artifacts that arise during cryocooling (Fraser *et al.*, 2011 ▸), as well as opening up the possibility of harnessing more information about protein dynamics.

Although multi-crystal experiments have been carried out in the past for very radiation-sensitive samples such as viruses (Abrescia *et al.*, 2004 ▸; Ji *et al.*, 2009 ▸), the high brilliance of third- and fourth-generation synchrotrons and X-ray free-electron laser (XFEL) sources has propelled the recent rapid development of serial crystallography. The serial approach is absolutely required at XFELs, where only one diffraction pattern is collected from the very short X-ray pulse (tens of femtoseconds) before the sample disintegrates as plasma (diffraction before destruction; Chapman *et al.*, 2011 ▸; Neutze *et al.*, 2000 ▸). However, the limited availability of XFEL time in conjunction with the continuous developments in hardware at synchrotrons, including newer faster detectors as well as bright microfocus beams, has resulted in increased interest of the structural biology community in serial millisecond crystallo­graphy. This interest has driven the development of novel and increasingly user-friendly sample-delivery methods (Yamamoto *et al.*, 2017 ▸; Sui & Perry, 2017 ▸). For monochromatic X-ray diffraction work, these platforms have included raster-scanning or small-wedge data collection from small crystals mounted in micromeshes (Zander *et al.*, 2015 ▸), the use of low-background fixed targets (Owen *et al.*, 2017 ▸; Roedig *et al.*, 2016 ▸; Zarrine-Afsar *et al.*, 2012 ▸; Mueller *et al.*, 2015 ▸; Huang *et al.*, 2015 ▸; Coquelle *et al.*, 2015 ▸; Baxter *et al.*, 2016 ▸; Axford *et al.*, 2016 ▸; Schubert *et al.*, 2016 ▸; Doak *et al.*, 2018 ▸), conveyor belts coupled to acoustic injectors (Roessler *et al.*, 2013 ▸) or to liquid dispensers (Beyerlein *et al.*, 2017 ▸), high-viscosity injectors (Weinert *et al.*, 2017 ▸; Kovácsová *et al.*, 2017 ▸; Sugahara *et al.*, 2015 ▸), quartz capillaries (Stellato *et al.*, 2014 ▸) and more recently microfluidic flow devices (Monteiro *et al.*, 2019 ▸). Liquid jets, such as those used to deliver samples at XFELs, are not suitable for millisecond crystallography owing to the very short residency time of the crystals in the X-ray-interaction region as a result of the fast fluid flow.

Sample-delivery methods for SSX are not ‘one size fits all’. As shown in Table 1 ▸, which summarizes the different serial crystallography experiments performed at monochromatic X-ray sources reported to date, data-collection times and sample consumption vary widely and depend greatly on the delivery method used and the nature of the protein-crystal slurry (crystal quality, concentration and monodispersity). It is thus vital to consider which experimental design is most suitable for the sample and biology in question. Clearly, high-viscosity injectors and fixed targets promote low sample consumption and are amenable to light-based pump–probe time-resolved experiments. Nevertheless, they require handling of the crystals during loading, which can be problematic for samples that are sensitive to, for example, humidity changes.

Microfluidics, on the other hand, open the possibility of probing the crystals in their original crystallization medium with minimal atmospheric exposure as well as with *in situ* diffusion of ligands. However, SSX in flow is still underrepresented. To date, only two experiments have been reported: one in which data were collected from a microcrystalline slurry flowing inside a simple quartz capillary (Stellato *et al.*, 2014 ▸) and one using an X-ray-compatible microfluidic device which employed 2D flow-focusing (Monteiro *et al.*, 2019 ▸). This underrepresentation arises from the complexity of the experimental design and the difficulty of fabrication of X-ray-compatible microfluidics.

When considering the choice of device and experimental design, several interconnected factors have to be satisfied for the experiment to be successful. The first consideration is signal to noise: while high photon flux is desired, this can quickly lead to damage and fouling of the microfluidic chip windows as a result of radiation-induced sample aggregation. Large crystals will increase the signal, but are undesirable for mixing and flow experiments owing to long diffusion times as well as an increased propensity for sample clogging. Secondly, the crystal residency time in the beam necessary to obtain a usable diffraction pattern determines the exposure time and the maximum flow rate that can be used. Too high flow rates lead to insufficient residency time in the X-ray beam and too slow flow rates lead to the damage of the sample, window fouling, the emergence of bubbles and possible clogging of the microfluidic device. Nevertheless, this report, along with the two previously reported SSX flow experiments (Stellato *et al.*, 2014 ▸; Monteiro *et al.*, 2019 ▸), shows that it is possible to satisfy all of these conditions. However, it is important to note that all three experiments were carried out on different beamlines, with different detectors, photon fluxes and beam sizes, and therefore that the design of the data-collection strategy had to be tailored to the local conditions. SSX experiments using *in situ* microfluidics are very efficient in terms of both sample consumption and data-collection time compared with other flow experiments, particularly those using free jets at XFELs. There are two major developments in this manuscript compared with the previously reported SSX experiments in flow: the first is the implementation of a 3D flow-focusing geometry, rather than just 2D, and the second is the development of a device-fabrication route which utilizes available and affordable 3D-printing methodology for fast and reproducible manufacture.

3D flow-focusing geometries, such as that shown in our 3D-MiXD device, yield a much more uniform sample velocity, owing to the central positioning of the sample in the parabolic flow profile (Wunderlich *et al.*, 2014 ▸), than that obtained with 2D focusing; in turn, this allows accurate X-ray radiation dose calculations and prevents sample damage and aggregation to the device walls. However, 3D flow-focusing microfluidic device fabrication is challenging, especially when X-ray compatibility is needed. Capillary-based co-flowing devices consisting of two concentric capillaries, a central one for the sample and an outer one for the focusing buffer, have been used to generate 3D-focused samples for droplet-based small-angle X-ray scattering (SAXS) experiments (Stehle *et al.*, 2013 ▸). More advanced geometries have been coupled to gas dynamic virtual nozzles (GDVNs) which generate free-standing liquid jets for serial femtosecond crystallography (SFX) experiments (Calvey *et al.*, 2016 ▸; Olmos *et al.*, 2018 ▸). However, as mentioned, liquid jets cannot be used in monochromatic SSX owing to the very short sample residency time in the beam. Flow-focusing X-ray-compatible capillaries fabricated to couple to a GDVN should allow the collection of SSX data *in situ*, but this has yet to be demonstrated. It is important to note that the manufacture of such mixers involves the cutting, polishing, centering and securing of concentric capillaries and therefore the geometry is limited by the sizes and wall thicknesses of the commercially available capillaries.

3D microfluidic devices are often manufactured by the layering and aligning of multiple patterned 2D layers, which is a labor-intensive process (Köster & Pfohl, 2012 ▸; Brennich *et al.*, 2011 ▸). 3D printing, in contrast, is a versatile and highly reproducible platform for microfluidic device production (Ho *et al.*, 2015 ▸; He *et al.*, 2016 ▸; Waheed *et al.*, 2016 ▸; Männel *et al.*, 2018 ▸) and has alleviated some of the manufacturing burden associated with generating full 3D microfluidics. However, 3D-printing resins are not X-ray-compatible, so for SFX experiments at XFELS the 3D-printed flow-focusing parts have to be coupled to GDVNs (Ishigami *et al.*, 2019 ▸). The 3D-MiXD that we describe here was designed to be easily manufactured using an inexpensive, commercially available 3D printer and requires a single post-printing step to seal the channels with X-ray-compatible foils. We also discuss the various aspects that are taken into consideration during its design.

## 2. Materials and methods   

### 2.1. Sample preparation and injection   

All solutions were filtered through a 0.22 µm filter and all plasticware was thoroughly dusted prior to crystallization. Hen egg-white lysozyme was obtained from Sigma–Aldrich (catalog No. 62971). 10 and 25 µm lysozyme crystals were obtained using a modified protocol for batch crystallization previously reported by Gorel *et al.* (2017 ▸). In brief, hen egg-white lysozyme at 30 mg ml^−1^ in 100 m*M* sodium acetate buffer pH 3.0 was mixed rapidly in a 1:1, 2:3 or 1:2 ratio with crystallization buffer (100 m*M* sodium acetate pH 3.0, 20% NaCl, 6% PEG 6000) to a total volume of 1.5 ml in a 2 ml Eppendorf tube. The tube was placed on a slowly rotating axis (10 min^−1^) overnight at room temperature. The protein:precipitant ratio as well as the speed of mixing influenced the final size of the crystals.

Aspartate α-decarboxylase (ADC) was expressed and purified as described previously (Monteiro *et al.*, 2012 ▸, 2015 ▸). In brief, His-tagged wild-type ADC was overexpressed using an *Escherichia coli* Δ*panD* Δ*panZ* (DE3) cell strain from the vector pRSETA-ADC-WT (Saldanha *et al.*, 2001 ▸) using an autoinduction protocol. Cells were isolated by centrifugation (10 000*g*, 15 min), resuspended in buffer *A* (50 m*M* potassium phosphate, 300 m*M* NaCl pH 7.4) containing 10 m*M* imidazole and mechanically lysed using a Constant Systems cell disrupter (137 MPa), and the lysate was cleared by centrifugation (30 000*g*, 45 min). DNase I (Roche; ∼0.5 mg per litre of culture) was added before purification by Ni–NTA affinity chromatography using buffer *A* containing 50 m*M* imidazole as the wash buffer and buffer *A* containing 250 m*M* imidazole as the elution buffer. The protein-containing fractions were combined, concentrated and buffer-exchanged into 50 m*M* Tris–HCl pH 7.5, 100 m*M* NaCl, 0.1 m*M* DTT using a HiTrap (GE) 5 ml desalting column. The protein was then concentrated to 25 mg ml^−1^. 25 µm microcrystals were obtained by batch crystallization. The protein solution was rapidly mixed with vortexing in a 1:3 ratio with crystallization solution (1.95 *M* ammonium sulfate, 100 m*M* citrate/disodium phosphate buffer pH 3.8) in a total volume of 1.5 ml in a 2 ml Eppendorf tube at 21°C. Crystals formed within 4 h. The size of the crystals was extremely sensitive to changes in the ammonium sulfate concentration and the ages of both the mother liquor and the protein. It is important to note that ammonium sulfate solution is deliquescent and can be used to maintain the relative humidity of the surrounding environment; it will undergo changes in concentration over time and so it was made fresh shortly before microcrystallization. The final crystal sizes for both proteins and qualitative size dispersions were determined by manual inspection using a microscope. Shortly before loop-loading, the microcrystalline slurries were concentrated twofold by removing half of the supernatant, leaving approximately a 30% volume fraction of settled crystals. The crystallization buffer was loaded into a 1 ml Hamilton gas-tight syringe, which was used to push the crystal slurry into the device using pulsation-free neMESYS syringe pumps (low-pressure module, 290N Cetoni) at a flow rate of 50 µl h^−1^.

### 2.2. Device fabrication   

The microfluidic device body was 3D-printed using an Asiga PICO 2HD 3D printer (385 nm UV LED, 37 × 37 µm voxels) in MF RTP1 resin designed by Resyner Technologies C.L. (brand name ‘3Dresyns’). The device design is shown in Supplementary Fig. S1. A detailed technical drawing can be found in the supporting information. 25 µm layers were employed. The burn-in time was 0.6 s and the exposure time for all subsequent layers was 0.4 s. Once printed, the chips were cleaned by sonication twice in ethanol for 2 min and dried with compressed air. The 3D-printed structure surface roughness was ∼3 µm (1.1% of the channel width). Two Kapton windows (7.6 µm thick; VHG Labs), one 3 × 8 mm and one 5.5 × 13.5 mm, were cut with a scalpel to seal the device from the front and the back, respectively. The windows were washed with ethanol, dried in air and glued onto the device using two-component epoxy glue (Araldite). Gluing was accelerated by placing the device at 60°C for at least 2 h. 1.09 mm outer diameter polyethylene tubing was fitted to the inlets and outlet and secured in place using two-component epoxy glue (UHU). The devices were tested for leaks by flowing 0.22 µm-filtered water through all of the channels.

### 2.3. Beamline characteristics and data-collection strategy   

Data were collected on beamline ID30A-3 at the ESRF. The X-ray energy was 12.8 keV and the beam size was 15 × 15 µm horizontal × vertical FWHM. The rotating fast shutter was used to provide intermittent X-rays to the sample, equating to cycles of ∼300 ms of X-rays on/off, to allow the dissipation of radicals. The average flux at 100% transmission was 2 × 10^13^ photons s^−1^. Data were collected at 45–50% transmission (0.9–1.0 × 10^13^ photons s^−1^). The EIGER 4M (Dectris) detector-to-sample distance of 135.88 mm at 12.8 keV X-ray energy allowed the collection of data to a resolution of 1.5 Å at the edge. 5 ms exposure times were used for each image, with the detector running at 200 Hz.

### 2.4. Data reduction and structure solution   

The diffraction images were integrated and merged using *CrystFEL* (White, 2019 ▸; White *et al.*, 2012 ▸) by invoking a combination of *MOSFLM* (Leslie & Powell, 2007 ▸), *DirAx* (Duisenberg, 1992 ▸) and *XDS* (Kabsch, 2010 ▸) or only *XGANDALF* (Gevorkov *et al.*, 2019 ▸) for indexing. *XGANDALF* was chosen as the sole indexing program for the final data sets. *Partialator* (*CrystFEL*) was used for merging (White, 2014 ▸). *R*_split_, CC*, signal-to-noise ratio (SNR), multiplicity and completeness were calculated using *compare_hkl* and *check_hkl* (*CrystFEL*). The merged data were imported into *CCP*4 (Winn *et al.*, 2011 ▸) using *CTRUNCATE* (French & Wilson, 1978 ▸; Padilla & Yeates, 2003 ▸), phased by molecular replacement using PDB entries 5mjj (for lysozyme; A. Meents, D. Oberthuer, J. Lieske & V. Srajer, unpublished work) and 1aw8 (for ADC; Albert *et al.*, 1998 ▸) as models in *MOLREP* (Vagin & Teplyakov, 2010 ▸) and refined using *REFMAC*5 (Murshudov *et al.*, 2011 ▸) within *CCP*4. Iterative rounds of manual model building and real-space refinement were performed using *Coot* (Emsley *et al.*, 2010 ▸) The diffraction-weighted dose was calculated using *RADDOSE*-3*D* (Zeldin *et al.*, 2013 ▸).

### 2.5. Computational fluid dynamics (CFD) simulations   

Three-dimensional CFD simulations were performed using the commercial software package *COMSOL* (v.5.2). All simulations were carried out at 293 K and 1 atmosphere. The 3D geometry of the channel was generated starting from a CAD file created using *AutoCAD* (Autodesk) and matched the final printed device as closely as possible. Two simulations were run, one using the viscosity and density of water for the calculation (8.90 × 10^−4^ Pa s and 1.00 g cm^−3^, respectively) and one using the viscosity of 5% PEG 6000 in water (3 × 10^−3^ Pa s; Holyst *et al.*, 2009 ▸) and the measured density of the mother liquor (1.09 g cm^−3^). The two simulations were virtually indistinguishable. The laminar water flow field was calculated at 293 K and 1 atmosphere by solving the incompressible Navier–Stokes equation under continuous flow conditions,
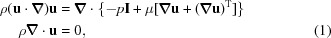
where ρ is the density of the fluid, *p* is the pressure, **I** is the identity matrix, μ is the dynamic viscosity of the fluid and **u** is the velocity field. The walls were defined as having no-slip conditions, the entrance length for the fluids was defined as 100 µm and the velocities were defined as the flow rates described in Section 3. The sample-inlet flow rate was 50 µl h^−1^. The focusing flow rate was 250 or 125 µl h^−1^ per channel for 10:1 or 5:1 flow-focusing ratios, respectively, equating to 500 or 250 µl h^−1^ total from two channels.

Once the laminar flow field had been calculated, the diffusion field of the sample was generated by solving the convection–diffusion equation
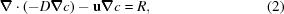
where *c* is the concentration, *D* is the diffusion coefficient (6.7 × 10^−10^ m^2^ s^−1^ for small-molecule diffusion and estimated at 2 × 10^−14^ m^2^ s^−1^ for 10 µm crystals by solving the Stokes–Einstein equation), **u** is the velocity field of the fluid and *R* is any source or sink of the species (*R* = 0 as there is no reaction of the solute).

Based on the laminar flow and concentration fields, the sample trajectory and local environmental changes were determined by using time-resolved particle tracing, where 500 particles were injected from the sample inlet and their positions were determined according to the local flow field. The time-resolved particle trajectories were calculated by solving
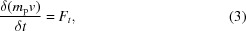
where *m*_p_ is the particle mass, *v* is the velocity, *t* is time and *F* is the Stokes drag force of the flow field.

All parameters, except for those listed here, were used from the *COMSOL*5.2 model library.

### 3.6. Data extraction for CFD simulations   

The sample flow lines were determined by the trajectories of the particles calculated in the particle-tracing module of the CFD simulation. The trajectories were plotted at the final time point of the simulation to show the full path of the particles through the channel. Images of these trajectories inside a 3D render of the full microfluidic device were plotted and exported directly from *COMSOL*. Histograms of the particle positions along the *Y* and *Z* coordinates (orthogonal to the flow direction, and perpendicular to and along the X-ray path, respectively) and the concentration field along the central *XY* and *XZ* planes of the device were plotted in *COMSOL* and exported as images (Supplementary Figs. S3 and S4).

## 3. Results and discussion   

### 3.1. Device design and fabrication   

3D printing is an easily accessible and very versatile technique for producing microfluidic devices. The printer used for this work, an Asiga PICO 2HD, is a digital light-projection (DLP) printer. DLP uses light to polymerize a photo-active resin into solid and water-tight structures. The device is built in layers, where each layer corresponds to one 2D exposure. The PICO 2HD printer can expose voxels of 37 µm and the practical minimum channel size printable depends on both the pixel size and the resin used (Männel *et al.*, 2018 ▸).

**Figure 1:**
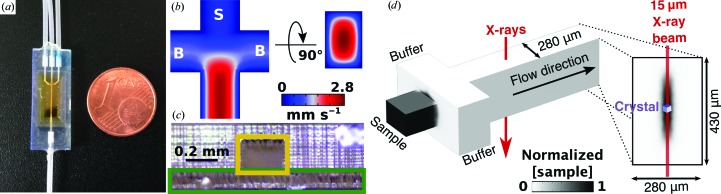
Chip geometry and design. (*a*) Final 3D-printed chip with tubing inlets and Kapton windows. The device is very small (8.88 × 23.10 × 2.32 mm) and has three inlet channels (two outer channels for the buffer and one central channel for the sample; top of image) and one waste outlet (bottom of image). The central device area is tapered to yield a thin, open central channel (430 × 280 µm vertical × horizontal; Supplementary Fig. S1) which is later sealed with Kapton HN foil. This is the X-ray-interaction region. (*b*) Schematic drawing. Two orthogonal central planes of the device showing the sample speed as calculated from computational fluid dynamics (CFD) simulations. The sample flows from the central inlet (S) and is focused by the perpendicular buffer inlets (B). (*c*) Cut-through view of the device at the sample inlet, showing the centering of the sample channel (yellow box) in the chip body and the minimal printable bottom layer height (green box). The sample-inlet channel is shallower than the buffer channels (200 µm versus 430 µm in height; both are 280 µm in width), causing the buffer to fully surround the sample (sides, top and bottom), focusing the sample in the center of the stream. (*d*) Perspective view of the channels and flow-focusing region to scale showing the normalized sample concentration of the focused sample stream (CFD data) and the direction of the X-ray beam. The inset depicts the channel cross-section from the perspective of the flow direction, with the beam width and crystal sizes represented to scale.

The 3D-MiXD designed, fabricated and used in this work is shown in Fig. 1 ▸(*a*) (see also Supplementary Fig. S1 for more detailed drawings; the technical schematics have also been supplied as supporting information). The most important aspect of the device design was the introduction of a 3D flow-focusing geometry, where the sample is focused in the center of the channel by buffer or water. Using this approach, the in-flowing microcrystalline slurry is fully surrounded by a carrier medium (buffer or water) before entering the main channel for X-ray data collection. Therefore, the sample flow is separated from the device walls and windows by a buffer layer, helping to avoid device fouling [Figs. 1 ▸(*c*) and 1 ▸(*d*)].

Pressure-driven flows in microchannels lead to a parabolic flow profile, with the liquid in the center of the channel flowing faster than that next to the walls, where the speed is virtually zero [no-slip condition; Fig. 1 ▸(*b*)]. As the X-ray dose experienced by a crystal is proportional to its residency time in the X-ray beam, crystals receive a higher absorbed dose when flowing next to the walls than at the center of the liquid stream, leading to damage to the protein as well as to the device. By using 3D flow-focusing to center the crystal flow in the 3D-MiXD, a much more uniform crystal speed is obtained, minimizing flow dispersion, and thus the X-ray dose per crystal is also more homogeneous and accurately calculable. This clearly defined dose is extremely important in serial crystallography experiments, as one of the main reasons for embarking on serial data collection is to obtain low-dose, un­damaged room-temperature data sets.

At the high X-ray photon fluxes used to maximize the signal-to-noise ratio and minimize the exposure time necessary to obtain a diffraction pattern, X-ray damage to the device must also be considered. The absorption of X-rays by the aqueous sample leads to the formation of photoelectrons, which in turn cause the degradation and aggregation of both sample and buffer components. This has been well described for SAXS measurements, as sample aggregation significantly alters the measured signal and is very difficult to model and deconvolute from the sample signal. For SAXS, flow-focusing conditions are already offered on several beamlines and are routinely employed to reduce sample damage and aggregation (Martel *et al.*, 2012 ▸; Round *et al.*, 2015 ▸; Jeffries *et al.*, 2015 ▸). For SSX, this change in background is not as crucial as the background is calculated locally (around each diffraction peak) during integration. Nevertheless, if the sample is allowed to touch the X-ray window, damaged material can rapidly aggregate and deposit. Besides an increase in background, fouling of the device windows can rapidly lead to complete disruption of the sample flow as the aggregate grows. When using 3D flow-focusing, as in the 3D-MiXD, the highly X-ray-sensitive sample is fully enclosed inside the buffer sheath [Figs. 1 ▸(*c*) and 1 ▸(*d*), Supplementary Figs. S3 and S4]. The buffer components at the wall will still experience high X-ray doses, but since they have a much lower aggregation propensity than proteins, fouling and aggregation are minimized (Kirby *et al.*, 2016 ▸; Kuwamoto *et al.*, 2004 ▸). In this experiment, no specialized additives were added to the buffer sheath and no significant increase in background or noticeable aggregation products were observed (Supplementary Fig. S3). When operated under stable flow conditions with no inclusion of air bubbles (from buffer degassing or from the sample-loading step), the 3D-MiXDs sustained more than 8 h of uninterrupted data collection.

The 3D flow-focusing geometry was incorporated into the 3D-MiXD through a simple cross-shaped junction (Fig. 1 ▸ and Supplementary Fig. S1). The main channel feeds into a cut-through cross, which begins the X-ray-interaction region. The main channel cross was sealed with two thin (7.6 µm) Kapton windows after additive manufacturing (3D printing). In order to achieve a sufficiently high signal-to-noise ratio from sample diffraction, the aqueous layer traversed by the X-ray beam (buffer/sample) had to be minimized. Therefore, a screening of printing parameters was carried out to define the thinnest 3D-printable channel height. The channel height is dependent on two factors: the smallest enclosed channel that can be successfully printed (sample inlet) and the minimum printable top/bottom layer heights.

For stability, the device was printed horizontally (Supplementary Fig. S1, top view), as all other printing directions yielded deformed channels from the floating-layer effect. This effect occurs when features in new layers being printed have no previously polymerized material to attach to and is especially problematic for thin layers. This effect is usually overcome by the addition of support structures to the design, which are removed after 3D prinitng. For the 3D-MiXD, the introduction of such support structures is undesirable as it would be virtually impossible to remove them from the channels without deforming them or greatly increasing the surface roughness. Therefore, thin floating layers (Supplementary Fig. S1, blue areas) were minimized by a combination of optimization of the design coupled with determination of the correct 3D-printing direction. The smallest printable enclosed channel was determined to be 200 × 280 µm (vertical × horizontal). Using the commercially available printer and resins described here, smaller channels were always found to be blocked by resin, which could not be flushed out. It is important to note that higher resolutions can be achieved using other setups, including customized 3D-printers as recently reported (Gong *et al.*, 2017 ▸).

Crystals of sizes up to 30 µm (the largest size tested) flowed without clogging during the course of our full experiment through the 3D-MiXD inlet channel. This is an important aspect as clogging is one of the greatest challenges for microcrystalline flow experiments (Wyss *et al.*, 2006 ▸; Dressaire & Sauret, 2016 ▸). The crystal slurries were not filtered to avoid damage to the crystals. Instead, optimized microcrystallization protocols (see Section 2) were developed to obtain highly monodisperse samples. The minimum top and bottom layer heights for the inlet channels were determined by the minimum burn-in layer height [Fig. 1 ▸(*c*), Supplementary Fig. S1, red] as this is the first layer exposed during fabrication and is responsible for attaching the growing device to the build platform. It is usually exposed for longer compared with the subsequent layers and is therefore thicker. For our chosen resin, this layer height was ∼130 µm. In order to center the sample inlet into the buffer channels to obtain a stable 3D flow-focusing geometry, a top layer of similar thickness was included, giving a final device thickness of 430 µm [Fig. 1 ▸(*b*)].

### 3.2. 3D-MiXD beamline integration   

**Figure 2:**
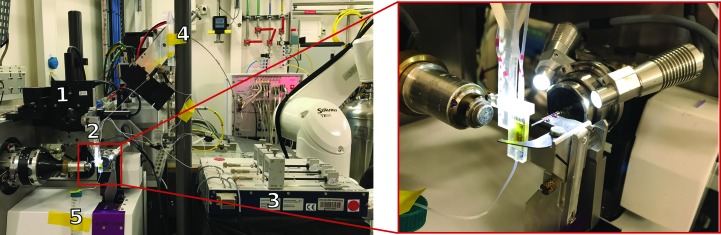
The 3D-MiXD device on beamline ID30A-3 at the ESRF. Overview of the setup showing the chip mounted and aligned with the X-ray-interaction region. The alignment was performed using a high-precision *XYZ* motor stage (1). The 3D-MiXD was mounted on a specially designed 3D-printed holder (2; enlarged in the inset). The liquid flows were controlled using high-precision syringe pumps (3); the microcrystalline slurry sample was loaded onto a Teflon loop (4) and waste was collected from the outlet (5).

The 3D-MiXD chip inlets and outlet were directly attached to polyethylene tubing. The minute footprint of the device (8.88 × 23.10 × 2.32 mm) allowed fast and easy setup on beamline ID30A-3 (MASSIF3) at the ESRF and should be compatible with most macromolecular crystallography beamlines without disturbing the standard diffractometer setups. Fig. 2 ▸ shows the final setup at MASSIF3, with the device aligned with the X-ray-interaction region (Fig. 2 ▸, inset). The small device footprint allowed the use of the standard beamstop, beam-cleaning capillary and apertures. Therefore, other than the introduction of a secondary set of motors for the alignment of the chip (‘1’ in Fig. 2 ▸), no further modifications to the beamline were needed, making it possible to obtain multiple data sets during a standard 24 h beamtime slot. The 3D-MiXD was secured onto a 3D-printed holder (‘2’ in Fig. 2 ▸) designed to interface with the motorized alignment stage. The flow of the sample and buffers were controlled using pulsation-free high-precision NeMESYS syringe pumps (‘3’ in Fig. 2 ▸).

The crystals were loop-loaded into a thin Teflon tube of 0.33 mm internal diameter coiled in a downward spiral into the device (‘4’ in Fig. 2 ▸). Up to a total of 500 µl of sample could be loaded, which is sufficient to run a 10 h experiment. The hydrophobic and lipophobic nature of Teflon minimizes crystal aggregation at the surface, which aided the smooth injection of the sample. Nevertheless, the loading procedure for further samples should be tested prior to experiments, as different samples may have different settling propensities. It is important to note that an antisettling device (typically used in XFEL sample-injection systems; Lomb *et al.*, 2012 ▸) could not be used for this experiment as even small movements of the tubing feeding the sample to the device led to visible fluctuation of the crystal stream. Fluctuation occurred only when the antisettling device was in use, but could also be observed if the tubing leading to the device was gently oscillated. This fluctuation continuously changes the sample:buffer flow-focusing ratio inside the chip and consequently the sample velocity. This change is highly undesirable as it makes it difficult to maintain a constant residency time of the crystals on the beam, as well as complicating the calculation of the crystal travel time from the sample inlet to the X-ray-interaction region. The fluctuation is very apparent owing to the very low sample flows that can be used with these devices and highlighted the importance of having access to an on-axis viewing system during the experiment. A simple waste reservoir was attached to the outlet of the device (‘5’ in Fig. 2 ▸).

### 3.3. Collection of diffraction data in flow   

To demonstrate the applicability of 3D-MiXD for SSX, diffraction data were collected from two soluble, globular protein systems: hen egg-white lysozyme (obtained commercially, for comparison to previous studies) and *E. coli* aspartate α-decarboxylase (ADC; expressed and purified in-house following a previously described protocol summarized in Section 2; Monteiro *et al.*, 2012 ▸, 2015 ▸). Lysozyme microcrystals were obtained using a slightly modified protocol to that previously described (Gorel *et al.*, 2017 ▸). Protocols for the microcrystallization of ADC were developed in-house and are fully described in Section 2. The two different proteins microcrystallize from significantly different precipitant mixtures: PEG 6000 with NaCl for lysozyme and ammonium sulfate for ADC. The different buffers presented a good opportunity to further test the robustness and stability of the devices and of the data-collection strategy. There was no significant increase in background from material deposition for data collected from the PEG-containing sample (Supplementary Fig. S2). Immediately before loading, settled microcrystalline samples were concentrated to approximately 30% volume fractions of crystals by removal of the supernatant and were gently resuspended.

All of the data presented in this study were collected from only two 3D-MiXD chips, one for each sample, and all data sets were collected under continuous chip operation. Each individual data set was collected at a single specific position along the device channel, *i.e.* the chip did not have to be translated during data collection since no material deposition on the channel walls was observed. The Kapton window was exposed for up to ∼90 min for a full data set at a single position without fouling of the window, disruption of the flow or significant increase in background (Supplementary Fig. S2), even if some darkening of the Kapton foil was visible.

**Figure 3:**
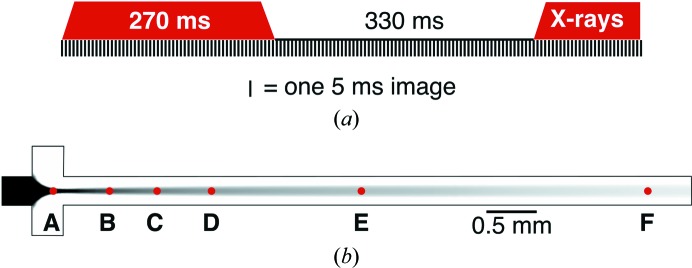
Data-collection strategy. (*a*) Schematic of X-ray exposure versus time with detector readouts (5 ms). (*b*) Center plane of the flow device showing the position of the X-ray data-collection points (red dots, labeled A–F according to Table 2 ▸) along the sample flow.

As shown in Table 2 ▸, complete and highly redundant data sets could be obtained in ∼60–90 min using only 50–70 µl of sample. Each data set was collected at a specific position on the chip [labeled A–F in Fig. 3 ▸(*b*)], maintaining the distance between the flow-focusing cross and the X-ray-interaction region constant during the data set. An accurately defined position of data collection relative to the flow-focusing cross (the mixing chamber) coupled with the uniform velocity of the channel-centered sample is extremely important for future time-resolved mixing experiments, as the time delay is defined by these two factors.

For the successful collection of diffraction data in flow, a compromise between X-ray exposure time and signal-to-noise ratio has to be achieved. The necessary exposure time is mainly dictated by the X-ray flux which, together with the X-ray focal spot size, yields the optimal crystal flow speed. A 10:1 (*v*:*v*) flow-focusing ratio between buffer and sample was chosen to provide a well centered crystal flow (see Supplementary Fig. S5). Flow rates of 50 µl h^−1^ for sample and 2 × 250 µl h^−1^ for buffer yielded an uninterrupted, smooth flow in the chip, which equated to speeds of ∼2.8 mm s^−1^ in the center of the channel as calculated from computational fluid dynamic (CFD) simulations [Fig. 1 ▸(*b*)]. The sample-flow stream thickness was calculated to be approximately 28 µm from CFD simulations in water (Supplementary Fig. S3) and is comparable to that measured experimentally at 26 µm (Supplementary Fig. S5). The sample-residency time in the 15 µm FWHM X-ray beam was ∼5.3 ms. Therefore, 5 ms frames were chosen for data collection to match the sample-stream speed and still provide full sample refreshment between images.

To avoid the formation of X-ray-induced bubbles, the fast shutter was used to turn the X-ray beam on and off during data collection by toggling its state every ∼0.3 s [Fig. 3 ▸(*a*)]. This results in half of the recorded images being empty, but these empty images compress well on the file system and can easily be discarded during data processing. This on–off X-ray beam pattern allowed the dissipation of X-ray-induced radiolysis products that can otherwise lead to the formation of bubbles in the channel as well as protein aggregates. These bubbles are thought to be H_2_ gas formed from the recombination of hydrogen radicals (Caër, 2011 ▸; Jonah, 1995 ▸). Bubbles generate hydrodynamic blockages, initially diverging and accelerating the liquid flow and, as a consequence, diminishing the residency time of the crystals in the X-ray beam and preventing high-quality diffraction patterns from being collected. If not removed, the bubbles can become large enough to prevent crystal flow and lead to clogging of the channel. Furthermore, the bubbles push the microcrystals towards the Kapton windows, causing the rapid deposition of material and window fouling.

Collection of data from flow-focused samples is a significant step towards the further development of such devices for *in situ* mixing applications. When employing a 10:1 flow-focusing ratio, a 50% jump in ligand concentration is achieved within the first 300 µm from the end of the mixing cross (Supplementary Fig. S3). We chose multiple positions along the channel for data collection to investigate the influence of the distance of the sample from the initial flow-focusing region on parameters such as the hit rate and diffraction resolution. As shown in Table 2 ▸, high-quality data can be collected at any position along the channel, either close to the flow-focusing region [0.5 mm; position B in Fig. 3 ▸(*b*)] or far away [6 mm; position F in Fig. 3 ▸(*b*)], and at constant speeds. According to Stokes–Einstein diffusion theory (4), where *D* is the diffusion coefficient, *k*_B_ is the Boltzmann constant, *T* is the temperature, η is the dynamic viscosity of the medium and *r* is the radius of the particle,
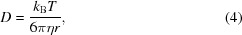
crystals on the micrometre scale have very long diffusion lengths, *i.e.* *D* for a 10 µm particle is very slow (2 × 10^−14^ m^2^ s^−1^). Therefore, under non-turbulent conditions, protein crystals remain essentially localized in the center of the fluid flow. This is corroborated by the near-constant and high indexable hit rate observed during data collection.

Data for the two proteins were collected at multiple points along the channel [Fig. 3 ▸(*b*)]. Seven ADC data sets were collected at distances varying between 0 and 6 mm from the flow-focusing cross. At a constant 10:1 flow-focusing ratio between the buffer and the crystalline slurry and at a total flow rate of 550 µl h^−1^, this 6 mm distance is equivalent to 2.14 s of sample-travel time from the start of the flow-focusing region. Two further lysozyme data sets [at 3 and 6 mm; positions E and F in Fig. 3 ▸(*b*)] were also collected for comparison. The data sets and their data-reduction statistics are summarized in Table 2 ▸. All of the data sets are available within the structure-factor file deposited in the PDB for the corresponding protein target (see Table 3 ▸ for the PDB codes).

The overall indexable hit rate for all ADC data sets was very high, varying between 13 and 40% of all X-ray ‘on’ images. The integration rates varied significantly depending on the indexing program used within *CrystFEL* (White *et al.*, 2012 ▸; White, 2019 ▸). Supplementary Table S1 shows a comparison of the hit rates and statistics obtained for three ADC data sets using different indexing-program combinations. It is clear that when using *XGANDALF* (Gevorkov *et al.*, 2019 ▸) the integration rate is considerably higher than when using a combination of *DirAx* (Duisenberg, 1992 ▸), *MOSFLM* (Leslie & Powell, 2007 ▸) and *XDS* (Kabsch, 2010 ▸). Therefore, *XGANDALF* was chosen as the sole integration software for all of the data sets in this manuscript. The hit rate did not decrease significantly along the length of the channel, instead fluctuating with the different data sets. This suggests a possible dependence on the sample or on crystal concentration gradients arising from the sample-loading step. The comparable hit rate of the 0.5 and 6 mm [positions B and F, respectively, in Fig. 3 ▸(*b*)] data sets highlights the efficient focusing of the crystals.

At the beginning of the flow-focusing region, where the crystal flow is not yet fully focused [0 mm data set, position A in Fig. 3 ▸(*b*)], a qualitative visual inspection of the frames during data collection indicated a very high hit rate, which was expected from both the slower speed of the crystals and the higher sample-to-buffer ratio (*i.e.* before the three inlet streams converge in the focusing channel, accelerating the fluid). This flow condition also leads to the emergence of multiple lattices in several of the patterns. As a result of this visual inspection, fewer frames were taken at this position (300 000). A high indexable hit rate of 40% was achieved, although it was still lower than expected from the initial visual inspection. More interestingly, a final resolution of 2.4 Å was obtained, which is significantly lower than that for the 0.5 mm ADC data set [position B in Fig. 3 ▸(*b*)], which had a similar number of indexed lattices (46 690 lattices, 2.00 Å resolution versus 53 539 lattices, 2.4 Å resolution). We cannot yet explain this observation, but believe it could be related to a possible increase in crystal rotation prior to complete 3D flow-focusing.

Two final experiments were run to further elucidate the performance of these devices. Firstly, a further ADC data set was collected 3 mm downstream of the flow-focusing region [position E in Fig. 3(*b*)] at a buffer:sample flow-focusing ratio of 5:1, with a correlated decrease in the overall flow rate to 300 µl h^−1^ (compared with the previous 10:1 ratio and 550 µl h^−1^). As expected, the 3D-focused flow thickness increased, reaching 48 µm (Supplementary Fig. S4). The decreased flow rate also yielded a decreased velocity of approximately 1.7 mm s^−1^ in the center of the channel and, in theory, an approximately doubled residency time in the beam for each crystal. The indexable hit rate did not increase under these conditions, showing that the number of hits is mostly defined by the sample concentration rather than the flow-focusing ratio or the speed of the sample inside the chip (up to 5 mm s^−1^). The increase in X-ray absorbed dose under these flow conditions (from decreased velocity) is undesirable, so data should always be collected at the maximum sample speed that still yields sufficient residency time with minimized X-ray damage and good diffraction patterns.

Secondly, two lysozyme data sets from crystals of similar size to those of ADC (25 µm) were also collected at 3 and 6 mm [positions E and F in Fig. 3 ▸(*b*)] and at 10:1 flow-focusing ratios for purposes of comparison. Both data sets were of comparable resolution to those from ADC. The indexable hit rates obtained were also similar to the ADC data sets. Therefore, the data collection was not affected by a change in the sample (although we note that both crystals were cuboid-shaped) or mother-liquor composition.

These observations show no clear dependence of hit rate on the flow ratios or flow speed (up to 5 mm s^−1^). Nevertheless, the behavior of the microfluidics and the obtainable indexable hit rate may change with, for example, the crystal morphology. Needles are especially difficult to handle in microfluidics and GDVNs in general. Furthermore, ideal microcrystalline volume fractions should be determined for smooth operation without clogging by pre-testing in flow in the final chip geometry. Such experiments with different crystal morphologies were beyond the scope of this initial study. It is important to highlight that all of the data sets collected here were highly redundant and were of similar resolution and quality. Each data set was collected in less than 1.5 h of continuous data collection, allowing the collection of multiple data sets along the continuous flow channel within a standard 24 h synchrotron beamtime.

**Figure 4:**
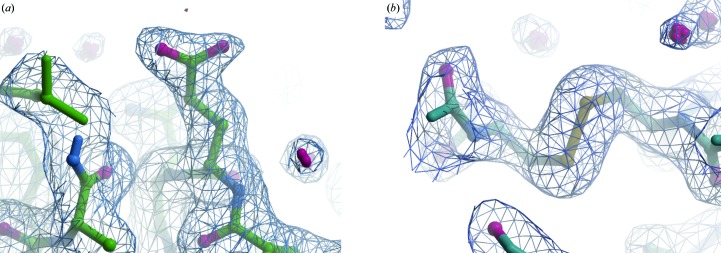
Refined ADC and lysozyme structures showing that residues susceptible to radiation damage remained intact. The 2*F*_o_ − *F*_c_ electron-density map (blue mesh) is contoured at 1 r.m.s.d. The *F*_o_ − *F*_c_ map (red/green mesh, contoured at 4 r.m.s.d.) is shown but no difference density is visible. (*a*) The surface glutamate (residue 96) of ADC. (*b*) The disulfide bond between residues 6 and 127 of lysozyme.

After integration, scaling and merging, two data sets, ADC at 3 mm and lysozyme at 3 mm, were solved to inspect the quality of the maps and of the overall protein structure (Table 3 ▸, Fig. 4 ▸). After phasing by molecular replacement using *MOLREP* (Vagin & Teplyakov, 2010 ▸) with PDB entries 5mjj and 1aw8, initial refinement using *REFMAC*5 (Murshudov *et al.*, 2011 ▸) yielded good electron-density maps that could be easily used for rounds of manual model building (Fig. 4 ▸). The overall X-ray dose (diffraction-weighted) was calculated with *RADDOSE*3*D* (Zeldin *et al.*, 2013 ▸) to be 74 kGy for ADC and 55 kGy for lysozyme. As expected, there was no evidence of radiation damage to the proteins during this room-temperature data collection, with both proteins showing intact disulfide bonds and carboxylate-containing side chains (Fig. 4 ▸). Furthermore, the high hit rates suggest that molecular hydrogen-bubble formation is well inhibited with the pulsed X-ray data-collection strategy.

## 4. Conclusion   

This study shows the capabilities of the combination of two powerful techniques: 3D printing and serial crystallography. By designing a microfluidic chip capable of centering protein microcrystals in a stable 3D liquid flow, serial synchrotron crystallographic data sets could be collected with low sample consumptions and fast collection times. This data-collection strategy satisfied the complex multi-dimensional problem presented at the beginning of this manuscript. The interplay between the 3D-MiXD geometry and achievable flow speeds, the X-ray beam size and peak brilliance, the intermittent X-ray exposure, crystal residency times and accessible detector frame rates prevented the formation of radiation-induced debris and gas bubbles, and allowed stable data acquisition over long periods of time. The 3D flow-focusing approach also brings the possibility of future time-resolved mixing experiments in which substrates or ligands can be added to the focusing buffer to initiate protein activity in the crystals. To date, all rapid-mixing time-resolved protein diffraction experiments have made use of open injector-based sample-delivery systems rather than closed microfluidic geometries. In the 3D-MiXD, the crystals never leave the fluidic device, minimizing crystal handling and offering a high degree of control over the environment that the crystals are exposed to (for example humidity), making the experiment suitable for sensitive crystals.

The microfluidic chip was designed to be amenable to additive manufacturing using a 3D printer and resins that are both commercially available, an important aspect in the development of tailored and user-friendly sample environments. The chips can be rapidly fabricated and easily used to test for flow conditions in-house prior to beamtime to aid the tuning of microcrystallization conditions. Microfluidics are not a ‘one size fits all’ solution and may not be suitable for all types of serial crystallography experiments. Nevertheless, here we show that with the correct chip design and experimental parameters, serial data can be efficiently collected in flow in native environments and at room temperature using widely available monochromatic X-ray sources with average microfocused beams and with minimal disruption of the beamline setup. With the minimized setup time, the robust nature of the chips and exquisite control over flow conditions, many data sets can be obtained in a single 24 h shift. This efficient collection scheme in combination with mixing geometry paves the way for recording molecular movies at synchrotrons in time-resolved SSX.

## Supplementary Material

PDB reference: aspartate α-decarboxylase, 6rxh

PDB reference: lysozyme, 6rxi

Supplementary figures and table. DOI: 10.1107/S2052252519016865/jt5040sup1.pdf

Technical drawings of the microfluidic device. All measurements are in millimetres. DOI: 10.1107/S2052252519016865/jt5040sup2.pdf

## Figures and Tables

**Table 1:** Serial crystallography experiments performed at monochromatic X-ray sources

Experiment	Protein targets	Setup type	Data-acquisition rate (Hz)	Crystal size (µm)	Indexing rate (%)	5000 indexed patterns	Sample use
This work	ADC†, lysozyme	In flow, 3D flow-focused	200	25	6–36‡	3–14 min	50 µl h^−1^
Monteiro *et al.* (2019 ▸)	Lysozyme	In flow, 2D flow-focused	100	10	1.7	50 min	50 µl h^−1^
Schulz *et al.* (2018 ▸)	FAcD§	Time-resolved pump–probe fixed target	12¶ ††	2–50	5–50	14–140 min	30 µl¶
Weinert *et al.* (2017 ▸)	Lysozyme, A_2A_R‡‡, MOSTO§§, TD1¶¶,	High-viscosity extruder	50	5–20 × 20–50	4–46	4–42 min	1.4 µl h^−1^
Martin-Garcia *et al.* (2017 ▸)	A_2A_AR†††, lysozyme, phycocyanin, Flpp3[Table-fn tfn10], proteinase K	High-viscosity extruder	10	5–20	1–5	3–14 h	5.8 µl h^−1^
Owen *et al.* (2017 ▸)	Myoglobin	Fixed target	23	60	15–33	11–24 min	65 µl
Beyerlein *et al.* (2017 ▸)	Lysozyme	Time-resolved capillary mixer–conveyor belt	25	6–8	27	12 min	36 µl h^−1^
Botha *et al.* (2015 ▸)	Lysozyme derivatives	High-viscosity extruder	10	10–15 × 30–60	9–30	28–93 min	1.3 µl h^−1^
Nogly *et al.* (2015 ▸)	Bacteriorhodopsin	High-viscosity extruder	14	20 × 3	0.4	25 h	2.4 µl h^−1^
Coquelle *et al.* (2015 ▸)	Lysozyme	Thin silicon wafer, nano X-ray beam rastering	2	20	33	2 h	500 nl high concentration
Stellato *et al.* (2014 ▸)	Lysozyme	In flow, 100 µm capillary	25	3 × 6	2.7	2 h	150 µl h^−1^

† *Escherichia coli* aspartate α-decarboxylase.

‡ X-ray ‘on’ images only; 3–18% including blank images.

§ *Rhodopseudomonas palustris* fluoroacetate dehalogenase.

¶ From personal communication with the authors.

†† Can reach 20 Hz with a faster detector.

‡‡ Thermostabilized adenosine A_2A_ G protein-coupled receptor.

§§ Molybdenum-storage protein.

¶¶ αβ-Tubulin–darpin complex.

††† Human A_2A_ adenosine receptor.

‡‡‡ Soluble fragment of the membrane lipoprotein Flpp3.

**Table 2:** Data-collection parameters All crystals were ∼25 µm in size, the exposure time per frame was 5 ms and the photon flux was kept constant at 0.9–1.0 × 10^13^ photons s^−1^ for all data sets. Data were collected at room temperature (293 K). Values in parentheses are for the outer shell.

	ADC							Lysozyme	
Structure	0 mm	0.5 mm	1 mm	1.5 mm	3 mm	6 mm	3 mm, slow	3 mm	6 mm
Travel time (s)	0	0.178	0.357	0.536	1.07	2.14	1.76	1.07	2.14
Position (Fig. 3 ▸)	A	B	C	D	E	F	E	E	F
Diffraction-weighted dose (kGy)	74	74	74	74	74	74	148	55	55
Total images	300000	900000	800000	651400	1000000	900000	1000000	600000	600000
X-ray ‘on’ images	135000	405000	360000	293300	∼450000	∼405000	∼450000	270000	∼270000
Total measuring time (min)	∼25	∼75	∼67	∼55	∼83	∼75	∼83	∼50	∼50
No. of indexed hits (X-ray ‘on’ images)	53539 [40%]	46690 [13%]	120312 [33%]	103146 [36%]	119807 [27%]	71353 [18%]	136656 [30%]	16295 [6%]	52233 [19%]
Unit-cell parameters									
Space group	*P*6_1_22							*P*4_3_2_1_2	
*a* = *b* (Å)	72.8							79.7	
*c* (Å)	219.0							38.6	
α = β, γ (°)	90, 120							90, 90	
Resolution (Å)	63.05–2.40 (2.48–2.40)	63.05–2.00 (2.08–2.00)	63.05–2.10 (2.18–2.10)	63.05–2.10 (2.18–2.10)	63.05–2.00 (2.08–2.00)	63.05–1.90 (1.96–1.90)	63.05–1.90 (1.96–1.90)	56.36–2.00 (2.08–2.00)	56.36–1.90 (1.96–1.90)
Total reflections	31681700 (2192074)	52857878 (3747759)	63922315 (4475872)	54029720 (3798513)	145874597 (10341162)	74221907 (5248590)	153552703 (10845138)	5547305 (389619)	21051552 (1472406)
Unique reflections	14336 (1376)	24287 (2374)	21070 (2045)	21070 (2045)	24287 (2374)	28182 (2756)	28182 (2756)	8876 (869)	10288 (998)
〈*I*/σ(*I*)〉	10.78 (1.54)	12.02 (1.78)	15.02 (2.52)	13.19 (2.03)	19.30 (2.73)	13.16 (1.69)	20.19 (2.54)	6.21 (1.83)	19.03 (2.95)
Completeness (%)	100 (100)	100 (100)	100 (100)	100 (100)	100 (100)	100 (100)	100 (100)	100 (100)	100 (100)
Multiplicity	2210 (1593)	2176 (1579)	3034 (2189)	2564 (1858)	6006 (4356)	2634 (1904)	5449 (3935)	625 (448)	2046 (1475)
*R*_split_	0.08 (0.72)	0.06 (0.62)	0.05 (0.40)	0.06 (0.47)	0.04 (0.40)	0.05 (0.72)	0.03 (0.56)	0.06 (0.57)	0.03 (0.40)
CC* (%)	100 (86)	100 (89)	100 (98)	100 (99)	100 (95)	100 (86)	100 (84)	100 (93)	100 (93)
Wilson *B* factor (^2^)	41.2	31.6	38.2	33.4	30.5	27.5	27.9	31.3	28.1

**Table 3:** Refinement statistics for ADC and lysozyme data sets

	ADC, 3 mm	Lysozyme, 3 mm
PDB code	6rxh	6rxi
Resolution (Å)	63.05–2.00 (2.05–2.00)	56.36–2.00 (2.05–2.00)
Total No. of reflections used	24203 (1744)	8437 (623)
No. of reflections for *R*_free_	1277 (89)	404 (29)
*R*_work_ (%)	15.2 (25.9)	15.8 (27.9)
*R*_free_ (%)	18.4 (32.2)	20.1 (24.9)
No. of atoms		
Total	2006	1093
Protein	1903	1042
Ligand/ion	7	2
Water	96	49
*B* factors (Å^2^)		
Protein	38.92	39.70
Ligand/ion	38.87	40.98
Water	45.64	44.63
Ramachandran plot (%)		
Favored	95.54	94.31
Allowed	4.46	5.69
Outliers	0	0
R.m.s. deviations		
Bond lengths (Å)	0.012	0.009
Bond angles (°)	1.787	1.584
Clashscore	1	1
