# Supplementary material for: 3D-MiXD: 3D-printed X-ray-compatible microfluidic devices for rapid, low-consumption serial synchrotron crystallography data collection in flow
Source: IUCrJ. 2020 Jan 16;7(Pt 2):207–19. doi: 10.1107/S2052252519016865 (PMC7055382; doi:10.1107/S2052252519016865)
Supplement: Supplementary file 2 [file m-07-00207-sup2.pdf]

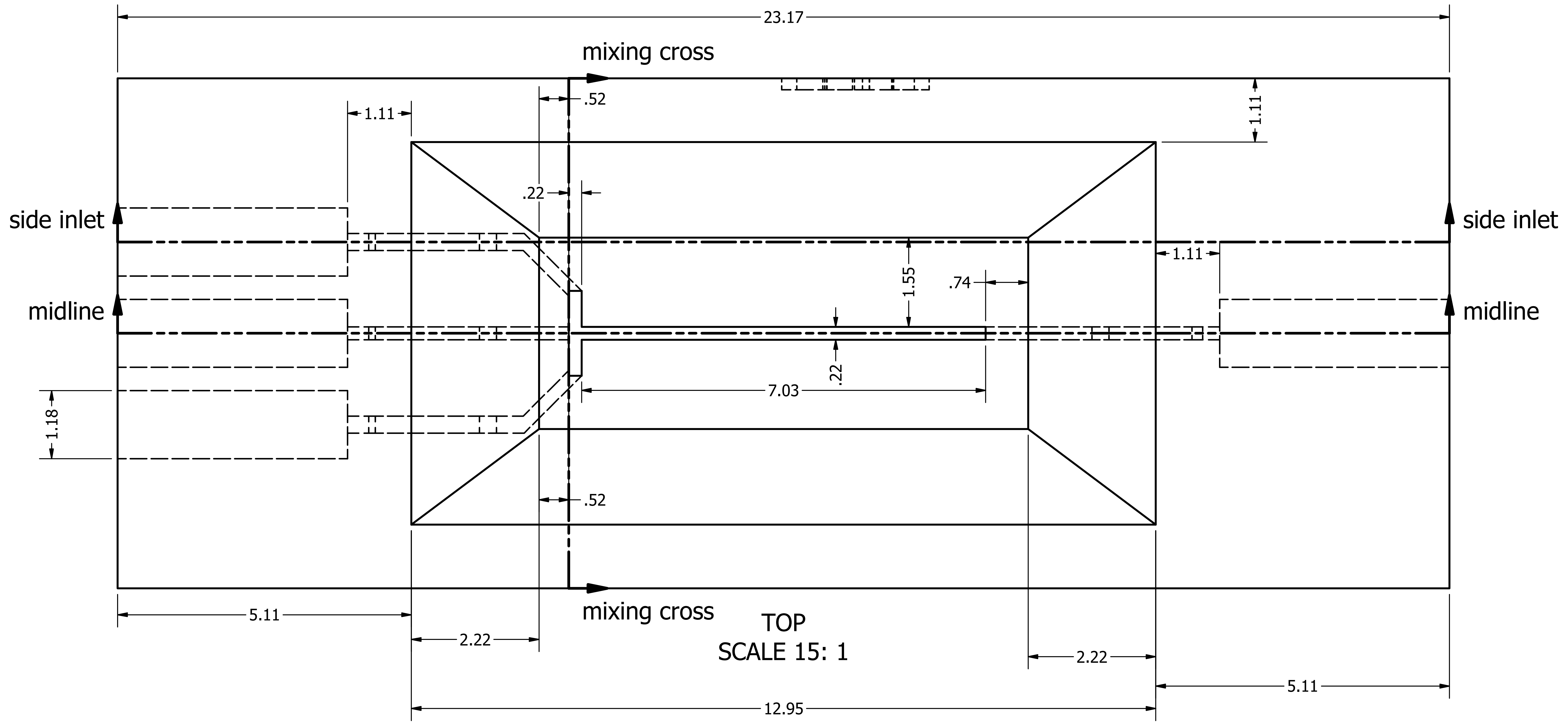

TOP
SCALE 15: 1

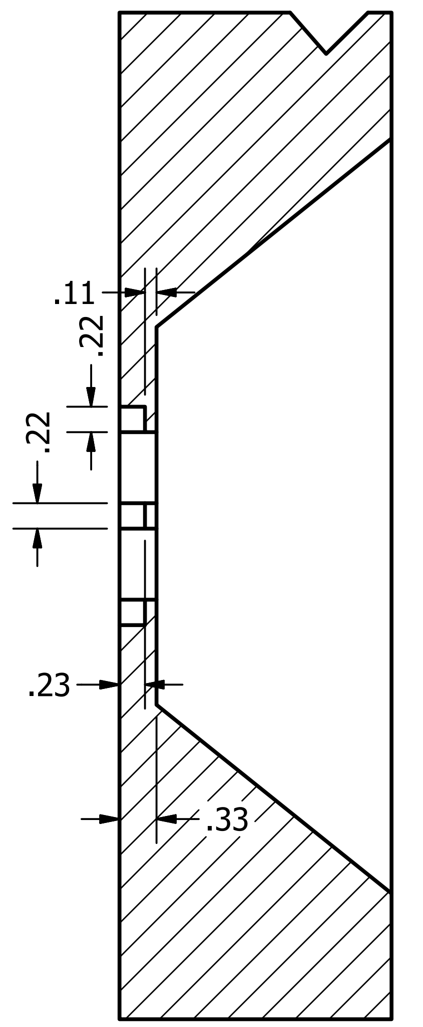

SECTION mixing cross
SCALE 15: 1

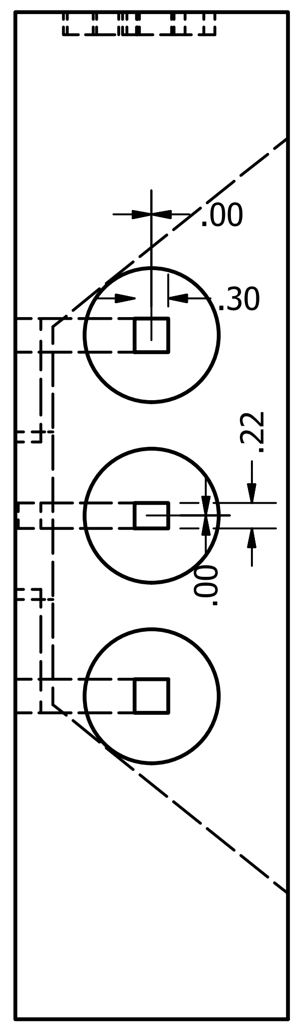

INLETS
SCALE 15 : 1

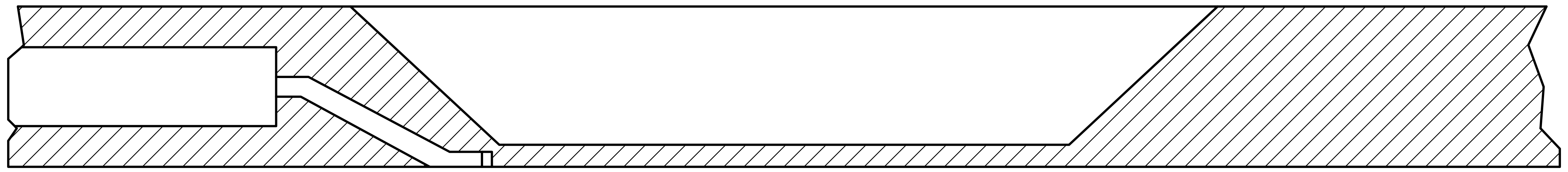

SECTION side inlet
SCALE 15: 1

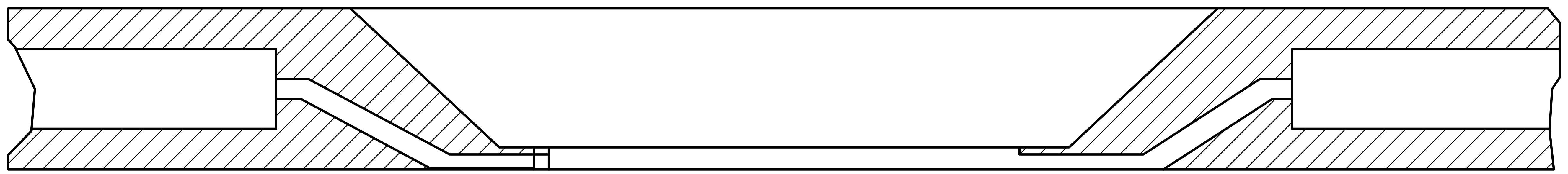

SECTION midline
SCALE 15: 1

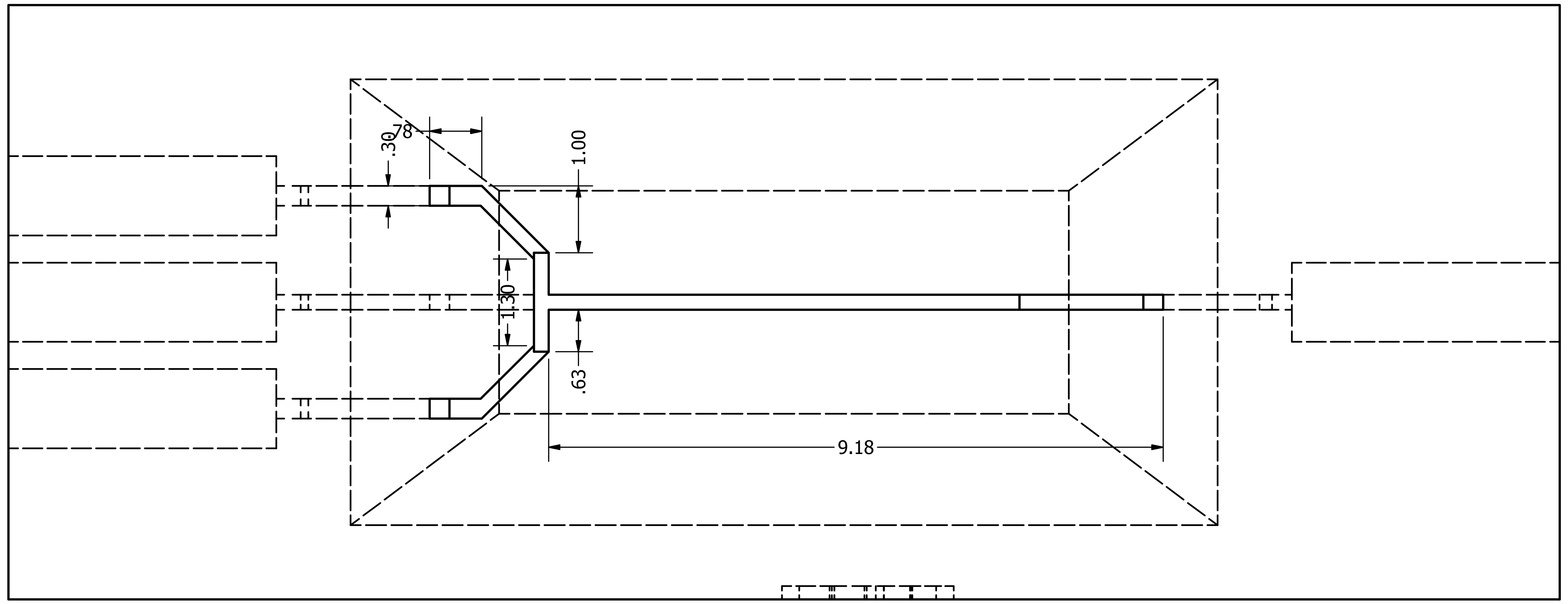

BOTTOM
SCALE 15: 1
