# Supplementary material for: 3D-MiXD: 3D-printed X-ray-compatible microfluidic devices for rapid, low-consumption serial synchrotron crystallography data collection in flow
Source: IUCrJ. 2020 Jan 16;7(Pt 2):207–19. doi: 10.1107/S2052252519016865 (PMC7055382; doi:10.1107/S2052252519016865)
Supplement: Supplementary file 1 [file m-07-00207-sup1.pdf]

IUCrJ

Volume 7 (2020)

Supporting information for article:

3D-MiXD: 3D-printed X-ray compatible microfluidic devices for rapid, low-consumption serial synchrotron crystallography data collection in flow

Diana C. F. Monteiro, David von Stetten, Claudia Stohrer, Marta Sans, Arwen R. Pearson, Gianluca Santoni, Peter van der Linden and Martin Trebbin

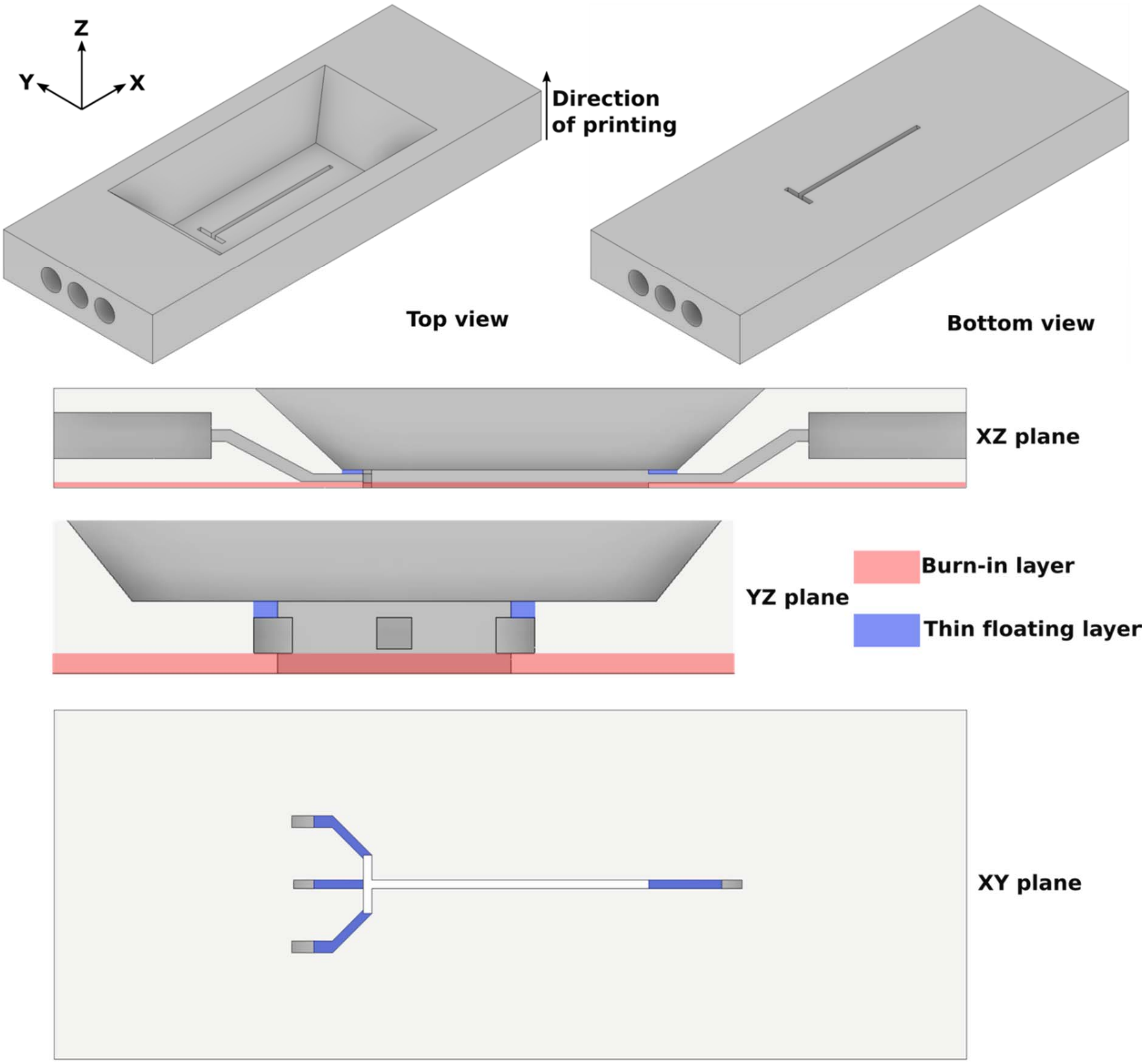

Figure S1 Overview of the 3D-MiXD geometry showing the direction of printing as well as the different layer types referred to in the main text. The XZ plane is a cut through the median line of the device. The YZ plane is a cut through at the flow-focusing region, showing the inlet channels. The XY plane is a view from the bottom of the device. The device dimensions are 8.88 x 23.10 x 2.32 mm. Due to ongoing design updates and improvements we encourage interested readers to contact the corresponding authors for the latest 3D-CAD geometry files.

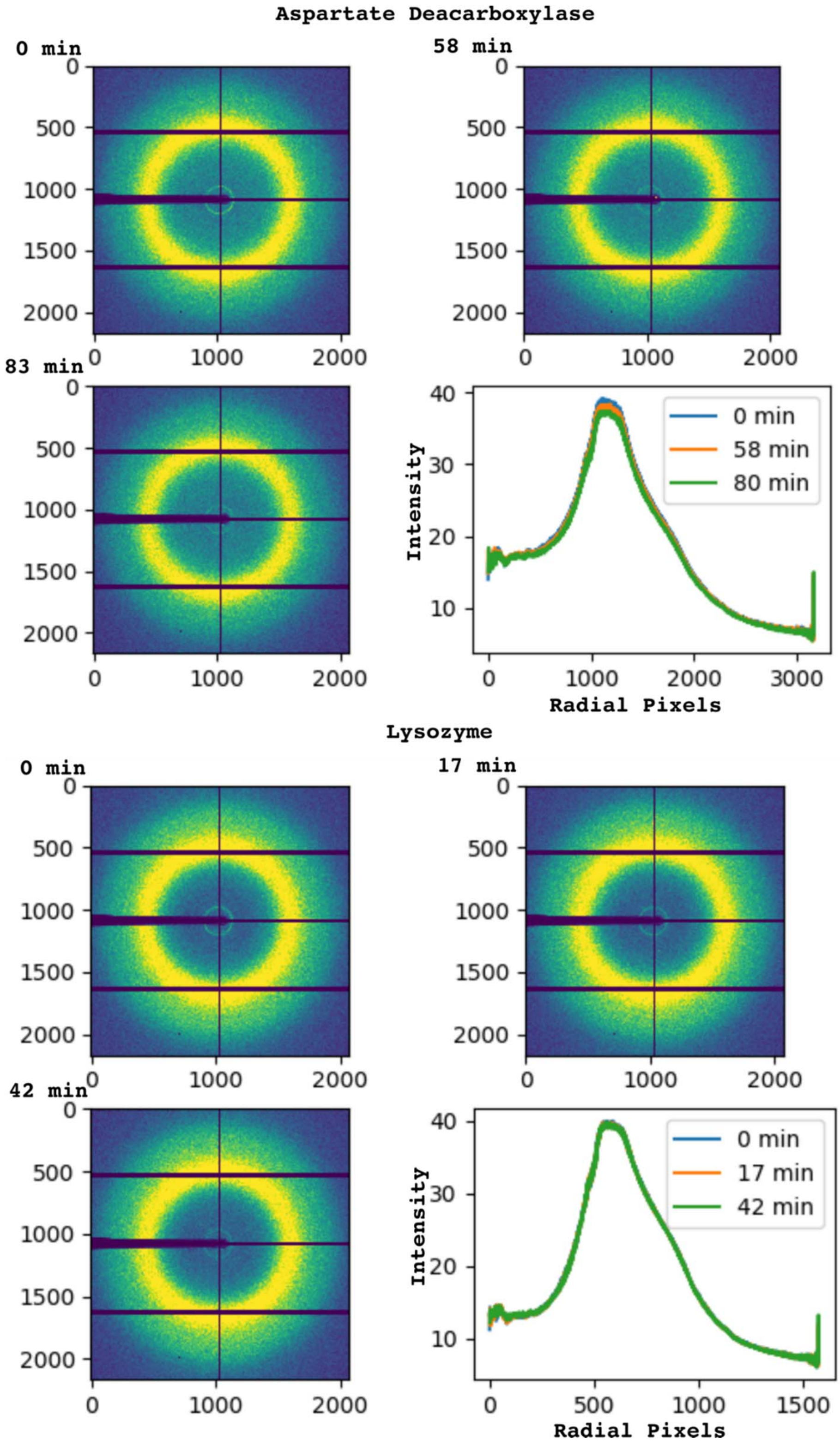

Figure S2 Radial integrations of the background (3D-MiXD and buffer) during data collection. 10 sequential images containing no visible hits were averaged and radially integrated at 3 different time points for both ADC and lysozyme (3 mm runs). No appreciable increase in background can be detected over the course of the data collection. No significant difference was detected in the background arising from either crystallization cocktail.

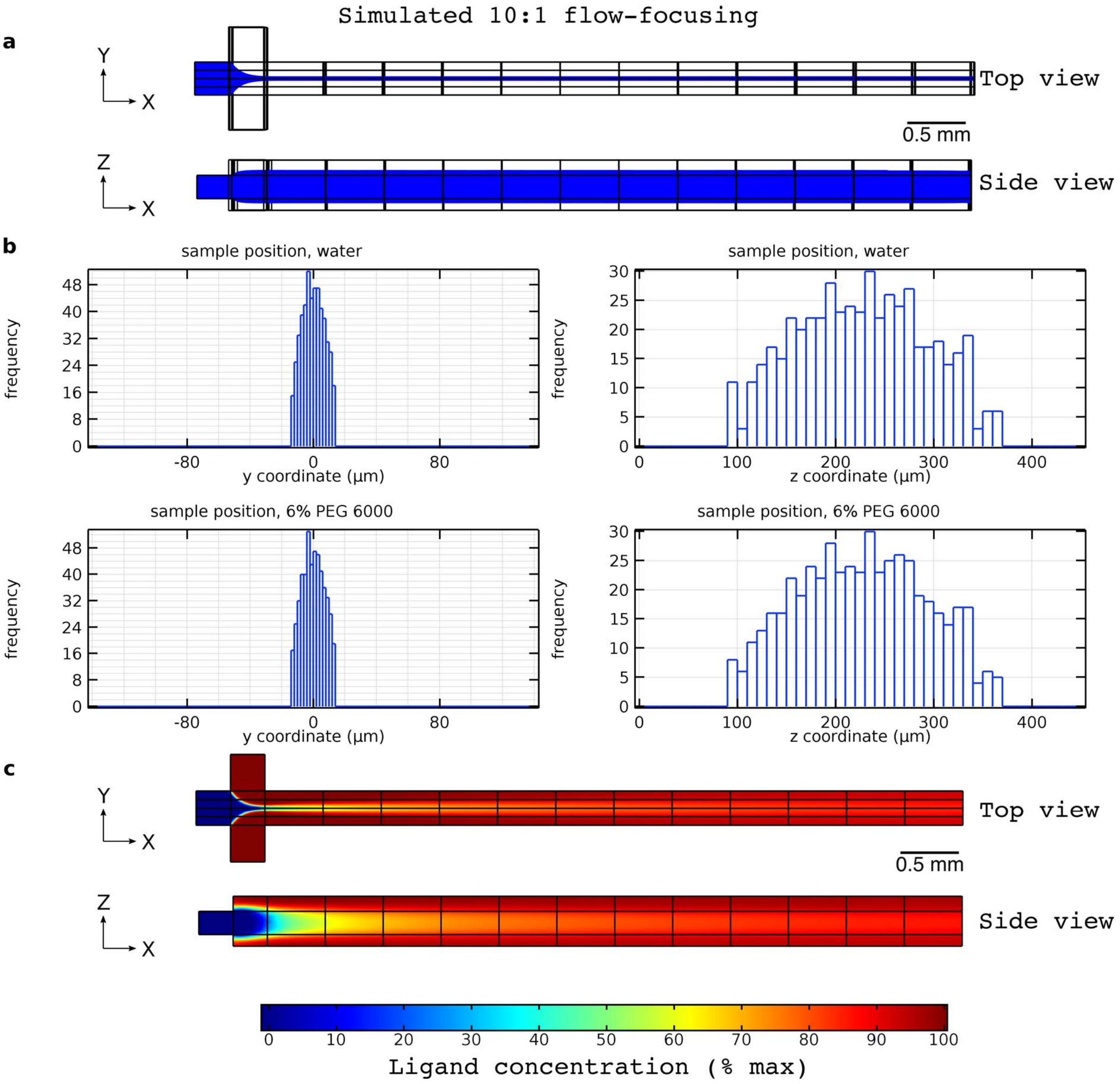

Figure S3 Computational fluid dynamic simulation of the device running at 10:1 flow focusing ratio. Two simulations were performed, one in water and a second using the viscosity of 5% PEG 6000 solution in water and the experimentally measured density of the buffer. a) 3D rendering of the sample flow inside the device (from particle trajectories) showing the 3D hydrodynamic flow-focusing which places the sample in the center of the channel, away from the walls. YX and ZX orientations are shown. b) histograms of the sample distribution (flow lines) for both simulations along the Y axis (XY plane in a) and the Z axis (ZX plane in a). The sample stream width is 28 µm. c) Concentration plots of a ligand diffusing in from the side channels (D = 6.7×10^{-10} m^2 s^{-1}). The central YX and ZX planes are shown. The average ligand concentration experienced by the sample is above 50% of the maximum value at the beginning of the main channel (after the flow-focusing cross). This change in concentration within the short mixing cross is sufficient to provide the initial jump for reaction initiation. The actual rate of reaction and concentration of intermediate species present will be dependent on the kinetics of the system as well as the concentration of the ligand.

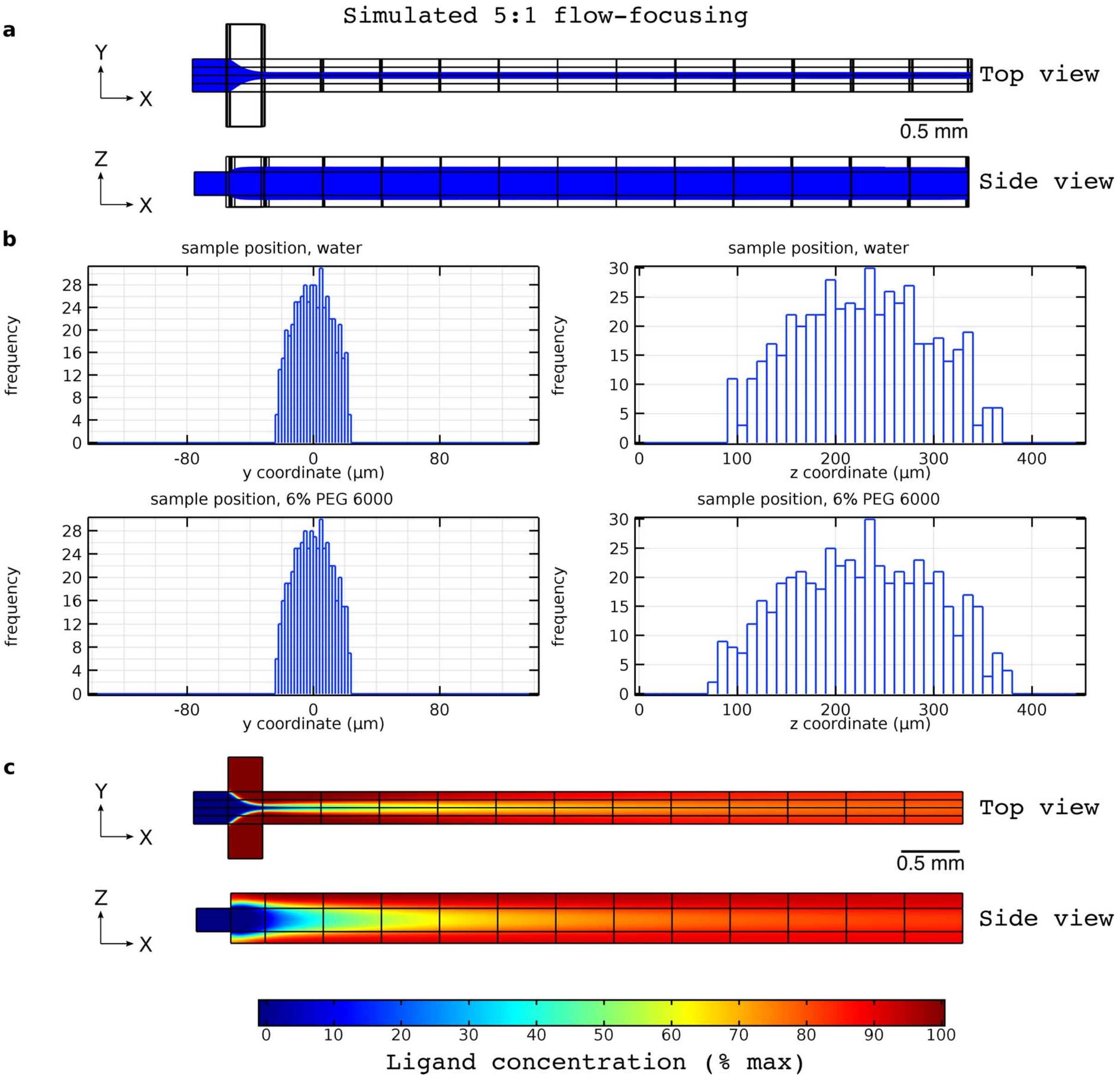

Figure S4 Computational fluid dynamic simulation of the device running at 5:1 flow focusing ratio for water and PEG (as per Figure S3). a) 3D rendering of the sample flow inside the device. YX and ZX projections. b) histograms of the sample distribution (flow lines) for both simulations along the Y axis (XY plane in a) and the Z axis (ZX plane in a). The sample stream width is 48 µm. c) Concentration plots of a small ligand diffusing in from the side channels ($D = 6.7x10^{-10}$ m^2 s^{-1}, central YX and ZX planes). The average ligand concentration is above 50% of the maximum value 0.5 mm into the main channel (after the flow-focusing cross).

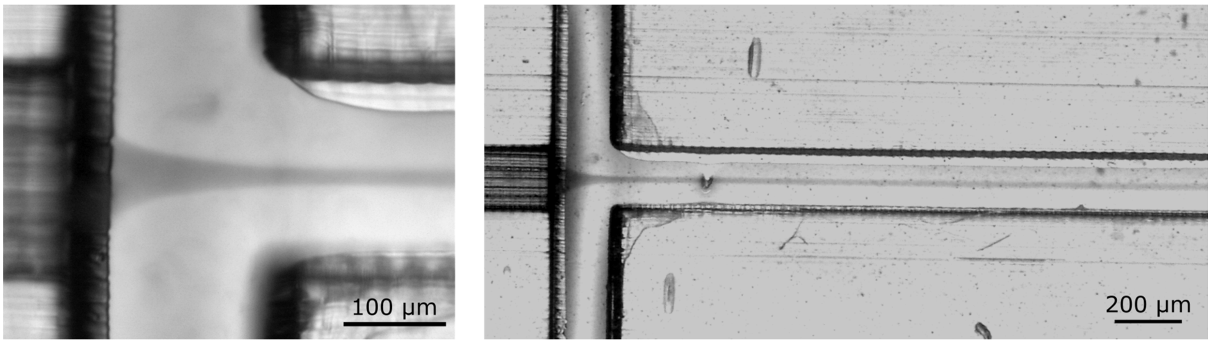

Figure S5 Microscopy image of the device running at 10:1 flow-focusing ratio in water. A dye was added to the fluid in the central channel (sample) to provide contrast. The flow-focused stream is ~26 µm.

Table S1 Comparison of algorithms for reflection integration within CrystFEL for 3 ADC datasets

All datasets were 100% complete across all resolution shells (with separate Fiedel pairs – Friedel's law was held true for the final merging of the data shown in the manuscript).

ADC 0p5mm 3 methods						ADC 0p5mm Xgandalf only					
Reflection distribution	288	0.7%	Dirax (third method)			Reflection distribution	46690	100%	Xgandalf		
	42639	98.6%	Mosflm (first method)				46690	100%	total		
	303	0.7%	XDS (second method)								
	43230	100%	Total								
Merging Statistics						Merging Statistics					
Resol. (Å)	N. Reflections	Red.	SNR <I/σ(I)>	Rsplit %	CC*	Resol. (Å)	N. Reflections	Red.	SNR <I/σ(I)>	Rsplit %	CC*
8.29	11579505	2610.9	26.80	3.65	1.00	8.29	11069799	2496.0	27.72	3.50	1.00
3.81	7367285	1670.2	20.31	4.49	1.00	3.81	7029049	1593.5	21.30	4.39	1.00
3.19	5398154	1227.1	12.17	7.62	1.00	3.19	5145385	1169.7	12.63	7.44	1.00
2.84	5127519	1172.8	8.08	11.68	1.00	2.84	4888183	1118.1	8.29	11.34	1.00
2.61	4846879	1100.8	5.57	18.42	0.99	2.61	4618198	1048.9	5.57	17.94	0.99
2.44	4572471	1041.3	4.38	23.88	0.98	2.44	4354182	991.6	4.25	24.57	0.98
2.31	4369797	991.6	3.79	27.27	0.97	2.31	4167095	945.6	3.57	29.57	0.97
2.20	4152524	950.0	2.93	36.10	0.96	2.20	3961310	906.3	2.69	38.72	0.95
2.11	4007327	916.8	2.20	48.01	0.94	2.11	3820364	874.0	1.94	54.17	0.92
2.04	3927799	886.0	1.54	70.82	0.86	2.04	3743405	844.4	1.32	83.18	0.83
ADC 1 mm 3 methods						ADC 1 mm Xgandalf only					
	1199	1.3%	Dirax (third method)				120312	100%	Xgandalf		

Reflection distribution	89455	97.5%	Mosflm (first method)			Reflection distribution	120312	100%	total		
	1111	1.2%	XDS (second method)								
	91765	100%	total								
Merging Statistics						Merging Statistics					
Resolution (Å)	N. Reflections	Red.	SNR <I/σ(I)>	Rsplit %	CC*	Resolution (Å)	N. Reflections	Red.	SNR <I/σ(I)>	Rsplit %	CC*
8.69	13843570	3613.6	28.31	3.29	1.00	8.69	13598616	3549.6	34.41	2.61	1.00
4.00	8742916	2294.1	22.71	4.03	1.00	4.00	8555853	2245.0	28.66	3.06	1.00
3.35	6607718	1739.3	13.09	7.34	1.00	3.35	6443076	1696.0	16.62	5.61	1.00
2.99	5939939	1559.9	7.99	12.43	0.99	2.99	5792544	1521.2	10.06	9.54	1.00
2.74	5638430	1484.2	5.48	18.08	0.99	2.74	5501031	1448.0	6.80	14.92	0.99
2.57	5320550	1405.3	3.83	27.07	0.98	2.57	5193756	1371.8	4.62	22.89	0.98
2.43	5091175	1343.0	3.05	35.97	0.96	2.43	4973750	1312.0	3.49	31.46	0.97
2.31	4839523	1286.1	2.64	40.46	0.95	2.31	4729150	1256.7	2.84	38.06	0.96
2.22	4714302	1240.0	2.08	52.43	0.91	2.22	4611724	1213.0	2.13	51.43	0.91
2.14	4569935	1203.2	1.87	56.10	0.97	2.14	4472374	1177.6	1.86	54.80	0.97
ADC 1.5 mm 3 methods						ADC 1.5 mm Xgandalf only					
Reflection distribution	1016	1.4%	Dirax (third method)			Reflection distribution	103146	100%	Xgandalf		
	72062	97.3%	Mosflm (first method)				103146	100%	total		
	952	1.3%	XDS (second method)								
	74030	100%	total								
Merging Statistics						Merging Statistics					
Resolution (Å)	N. Reflections	Red.	SNR <I/σ(I)>	Rsplit %	CC*	Resolution (Å)	N. Reflections	Red.	SNR <I/σ(I)>	Rsplit %	CC*
9.09	10014355	2987.6	24.77	3.61	1.00	9.09	10324651	3080.1	30.91	3.04	1.00
4.19	6400323	1934.2	21.41	4.26	1.00	4.19	6568765	1985.1	27.86	3.33	1.00
3.51	5190343	1573.8	13.49	6.79	1.00	3.51	5303087	1608.0	17.76	5.12	1.00
3.13	4118355	1245.3	8.11	12.04	0.99	3.13	4208225	1272.5	10.60	9.27	1.00
2.87	4102229	1240.8	5.53	18.66	0.99	2.87	4189697	1267.3	7.24	14.30	0.99
2.69	3879545	1184.2	3.94	26.78	0.98	2.69	3966609	1210.8	5.05	20.87	0.99
2.54	3753535	1133.3	2.91	36.88	0.96	2.54	3837165	1158.6	3.57	30.32	0.97
2.42	3597471	1090.1	2.44	44.24	0.94	2.42	3677647	1114.4	2.87	38.13	0.95
2.32	3456045	1051.4	2.11	52.10	0.91	2.32	3536556	1075.9	2.40	45.00	0.93
2.24	3369296	1015.5	1.73	63.93	0.89	2.24	3449639	1039.7	1.93	56.77	0.90
